# 54-year-old Woman with Chest Pain

**DOI:** 10.5811/cpcem.1666

**Published:** 2024-03-26

**Authors:** Zachary R. Wynne, Kami M. Hu, Laura J. Bontempo, J. David Gatz

**Affiliations:** * University of Maryland Medical Center, Department of Emergency Medicine, Baltimore, Maryland; † University of Maryland Medical Center, Department of Medicine, Baltimore, Maryland; ‡ University of Maryland School of Medicine, Department of Medicine, Baltimore, Maryland; § University of Maryland School of Medicine, Department of Emergency Medicine, Baltimore, Maryland

## Abstract

Chest pain is a common presentation to the emergency department (ED) that can be caused by a multitude of etiologies. It can be challenging to differentiate life-threatening conditions from more benign causes. A 54-year-old woman presented to the ED complaining of chest pain with dyspnea in the setting of recent blunt trauma. This case offers a thorough yet practical approach to the diagnostic workup of chest pain with dyspnea in the ED setting. The surprising final diagnosis and case outcome are then revealed.

## CASE PRESENTATION (DR. WYNNE)

A 54-year-old woman was brought into the emergency department (ED) by emergency medical services (EMS) for suspected intoxication. The EMS personnel found the patient in front of a convenience store who stated, “a fat lady fell on me.” She complained of diffuse chest pain, which was worse with deep breaths, and shortness of breath. Prehospital vital signs were unremarkable. No treatments were initiated, and the patient was transported to the ED. Upon arrival, the patient told the ED staff that earlier that day, someone had fallen on top of her. She denied head trauma and had no loss of consciousness. Not long afterward, she tried to drive home but began to feel lightheaded and pulled over at the convenience store. The patient complained of ongoing, continuous chest pain without radiation. There was no associated diaphoresis, paresthesias, or cough. The patient did complain of shortness of breath and again stated that her chest pain worsened with deep breathing. She denied any fevers or chills, abdominal pain, nausea, vomiting, diarrhea, urinary symptoms, neck pain, or back pain.

Her medical history was notable for chronic obstructive pulmonary disease (COPD) for which she used home oxygen. She also had a remote history of breast cancer previously treated with bilateral lumpectomies. Her home medications included an as needed albuterol inhaler, fluticasone nasal spray, and five milligram tablets of oxycodone as needed for chronic back pain. The patient reported a history of tobacco use, alcohol use (less than seven drinks a week), and occasional marijuana, cocaine, and ecstasy use. She was adamant she had not used any substances within the prior 24 hours.

On initial presentation, the patient’s vital signs were temperature 36° Celsius, heart rate 98 beats per minute, blood pressure 79/64 millimeters of mercury (mm Hg), respiratory rate 25 breaths per minute, and oxygen saturation 99% on room air. Her estimated body mass index was 21 kilograms per square meter. On examination, the patient was not diaphoretic and not in acute distress. Her pupils were equal and reactive without miosis. Her head showed no signs of trauma. Her neck had full range of motion with no cervical spine tenderness and no jugular venous distention. Her heart had regular rhythm, with borderline tachycardia, and with no murmurs. She had some left lateral chest wall tenderness without crepitus, deformity, or flail. Her lungs were clear to auscultation and symmetric bilaterally. On abdominal exam, she exhibited right upper and lower quadrant tenderness without rebound tenderness or guarding. Examination of the extremities showed no edema or tenderness. She was neurologically intact with a Glasgow Coma Scale score of 15 and no focal deficits.

**Image 1.**
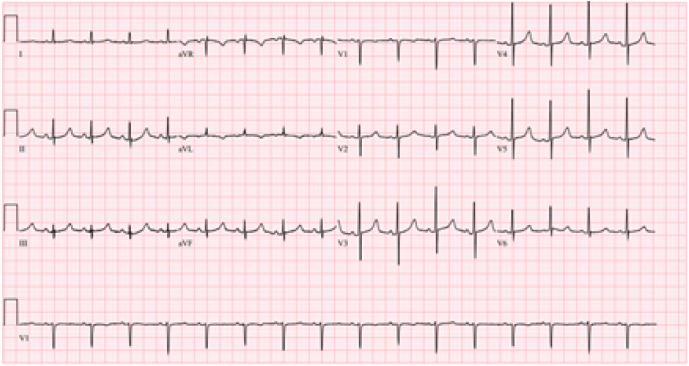
Electrocardiogram of a 54-year-old woman with chest pain.

**Image 2.**
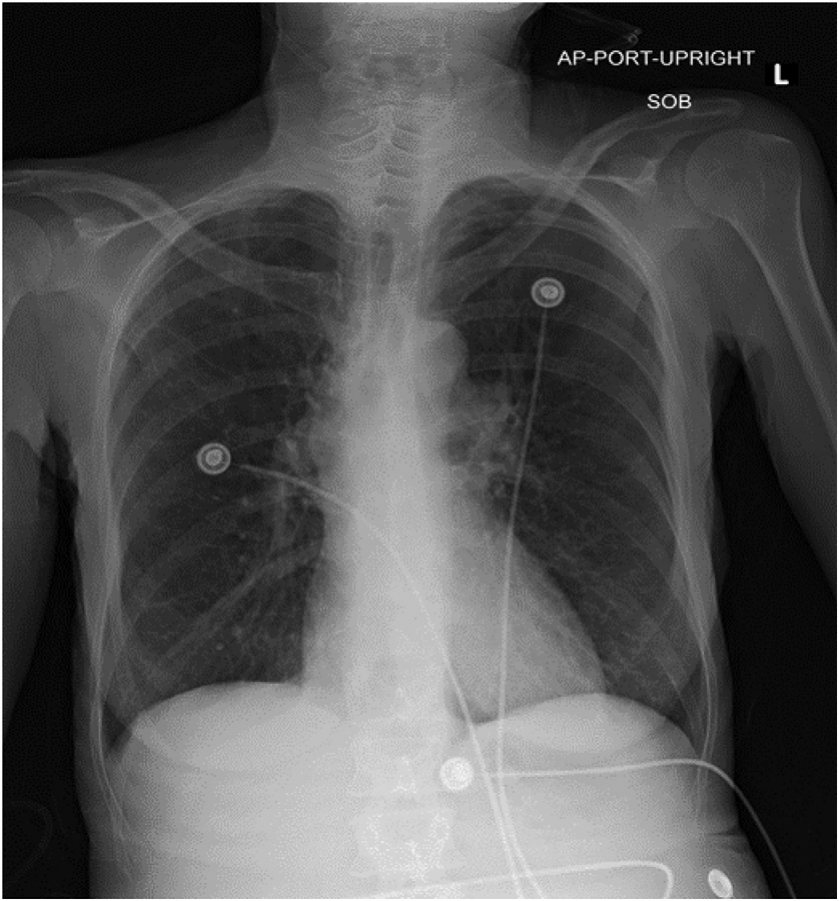
Chest radiograph of a 54-year-old woman with chest pain.

Laboratory studies were completed (Table 1). Complete blood count was notable for leukocytosis, mild anemia, and thrombocytosis. Basic metabolic panel was notable for an elevated blood urea nitrogen (BUN) and creatinine. Liver function tests were notable for an elevated aspartate aminotransferase (AST) with a normal alanine aminotransferase (ALT) level along with an elevated alkaline phosphatase with normal total bilirubin. Urinalysis was notable for pyuria, hematuria, and leukocyte esterase. Initial lactate was 3.8 millimoles per liter (mmol/L), which trended down to 3.1 mmol/L after crystalloid and vasopressor therapy initiation. Troponin biomarker was elevated along with NT-pro-B-natriuretic peptide and D-dimer. An electrocardiogram (ECG) and a chest radiograph (CXR) were obtained as well (Images 1 and 2).

**Table 1.** Initial laboratory results of a 54-year-old woman with chest pain.

Test	Patient value	Normal value
Complete blood count		
White blood cell count	23.0 × 10^3^/μL	4.0–10.0 × 10^3^/μL
Hemoglobin	11.8 g/dL	12.0–14.7 g/dL
Hematocrit	37.9%	36.0–45.0%
Platelets	467 × 10^3^/μL	166–362 × 10^3^/μL
Serum chemistries		
Sodium	139 mmol/L	136–145 mmol/L
Potassium	4.3 mmol/L	3.5–5.1 mmol/L
Chloride	100 mmol/L	98–107 mmol/L
Bicarbonate	22 mmol/L	21–30 mmol/L
Blood urea nitrogen	26 mg/dL	7–17 mg/dL
Creatinine	1.5 mg/dL	0.52–1.04 mg/dL
Glucose	295 mg/dL	70–99 mg/dL
Anion gap	17 mmol/L	
Calcium	9.5 mg/dL	8.6–10.2 mg/dL
Magnesium	2.6 mg/dL	1.6–2.6 mg/dL
Lactate - initial	3.8 mmol/L	0.5–2.2 mmol/L
Lactate - repeat	3.1 mmol/L	0.5–2.2 mmol/L
Hepatic studies		
Aspartate aminotransferase	55 U/L	14–36 U/L
Alanine aminotransferase	13 U/L	0–34 U/L
Alkaline phosphatase	144 U/L	38–126 U/L
Total bilirubin	0.4 mg/dL	0.3–1.2 mg/dL
Cardiac studies		
Troponin	0.9 ng/mL	<0.06 ng/mL
NT-proBNP	402 pg/mL	<300 pg/mL
D-dimer	5138 ng/mL FEU	<499 ng/mL FEU
Urinalysis studies		
White blood cell count	50 per hpf	0–5 per hpf
Red blood cell count	15–29 per hpf	0–2 per hpf
Leukocyte esterase	2+	Negative
Nitrite	Negative	Negative
Bacteria	Negative	Negative
Urine pregnancy test	Negative	Negative

*K*, thousand; *mcL*, microliter; *g*, gram; *dL*, deciliter; *mmol*, millimole; *L*, liter; *mg*, milligram; *u*, units; *ng*, nanogram; *pg*, picogram; *FEU*, fibrinogen equivalent units; *hpf*, high-power field; *NT-proBNP*, N-Terminal Pro-Brain Natriuretic Peptide.

The patient received intravenous (IV) fluid resuscitation with crystalloid. Concurrently, she was started on a norepinephrine infusion to maintain a mean arterial pressure (MAP) of greater than 65 mm Hg. As her MAP improved, the infusion was then discontinued. The patient was started on empiric antibiotics with piperacillin/tazobactam due to the concern for sepsis of unknown etiology as the cause of her hypotension. A test was subsequently ordered, and a diagnosis was made.

## CASE DISCUSSION (DR. HU)

Presented is a 54-year-old woman complaining of left-sided, pleuritic chest pain, lightheadedness, and shortness of breath after reported blunt trauma. There is concern from the accompanying medic that illicit substance use may be contributory, and the patient freely admits to substance use—but over 24 hours prior to symptom onset. There are no symptoms of recent illness or infection and, specifically pertinent to a trauma evaluation, no other pains and no hematologic issues.

The patient’s past medical history includes a diagnosis of COPD on supplemental oxygen at baseline, which might predispose her to a spontaneous or traumatic pneumothorax if she experienced a bleb rupture. Her history of breast cancer, presumably in remission, does give me pause—a recurrence of local or metastatic disease or secondary pathology such as a pulmonary embolism (PE) related to malignancy-associated hypercoagulability could potentially be related to her presentation as well. Her surgical history and medication list do not seem out of the ordinary for her provided history. As noted previously, she admits to occasional marijuana, methylenedioxymethamphetamine (commonly known as MDMA, “ecstasy,” or “molly”), and cocaine use, which can certainly cause coronary artery vasospasm and the symptoms associated with ischemia.

Her physical exam findings are most remarkable for hypotension with a quite narrow pulse pressure, borderline tachycardia, and tachypnea despite an otherwise normal cardiopulmonary auscultatory exam, including a normal saturation without her home oxygen. This raises concern for heart failure, volume loss, and cardiac tamponade. Aortic stenosis as a cause of the narrow pulse pressure is unlikely given that no murmur was auscultated on her examination. The patient is not only tender over her left chest wall, but on the right side of her abdomen as well, despite previously denying abdominal pain. This raises concern for intrabdominal pathology such as trauma-related liver injury. She has, notably, no other signs of injury.

The majority of the positive lab findings are non-specific, and their chronicity is unclear: the leukocytosis and thrombocytosis could be reactive or inflammatory, and the anemia is borderline. Her BUN and creatinine are elevated; however, even if it were acute, kidney injury does not narrow the differential diagnosis. The transaminitis is mild, and an elevated AST could mean injury to the liver, skeletal muscle, heart, kidney, or brain. Of note, the ALT, which is more specific to the liver, is within normal limits, as is the total bilirubin level, thus making primary liver pathology less likely. The most interesting lab abnormalities are the elevated troponin, natriuretic peptide, and D-dimer levels, as well as the lactate that does not clear with IV fluid administration.

Her CXR is generally unremarkable, without the classic (although uncommon) findings associated with PE such as Hampton hump, Westermark sign, parenchymal consolidation, Fleischner sign, or pleural effusion.^1^ The clear CXR eliminates a large hydro/pneumothorax or a flail chest as the etiology of her symptoms. Her ECG demonstrates a normal sinus rhythm, with normal intervals, normal axis, and an isolated T-wave inversion in lead aVL, eliminating acute coronary syndrome as the cause of her chest pain.

Primarily neurologic disorders are excluded from the differential diagnosis, given the lack of any central nervous system related physical exam findings and the presence of hypotension. Pathologies causing a secondary increase in intracranial pressure would be accompanied by a normal or elevated blood pressure and bradycardia rather than hypotension. Similarly excluded are isolated psychiatric disorders and milder illnesses such as gastritis, bronchitis, and muscle strains, which could be consistent with her complaints but are inconsistent with her vital sign abnormalities.

The patient has an elevated glucose and a mild anion gap, but a diagnosis of diabetic ketoacidosis does not explain the rest of her cardiac lab abnormalities. She is not bradycardic and has no other cardinal signs or complaints associated with hypothyroidism such as myxedema or hypothermia, nor does she have the hypertension, headaches, palpitations, or flushing usually associated with pheochromocytoma. Her electrolytes and blood pressure improvement with fluids make a diagnosis of adrenal insufficiency unlikely, while the lab findings of an elevated platelet count and normal total bilirubin exclude the hematologic emergency thrombotic thrombocytopenic purpura or other hemolytic anemias as a cause of her symptoms. A leukocytosis can be indicative of hematologic malignancy, but the white count elevation is too mild to be a cause of leukostasis leading to cardiac issues, and the leukemias are not typically associated with hypotension without other pathology.

While some toxicologic entities can cause hemodynamic instability, an elevated lactate, and cardiovascular lab abnormalities, it is unlikely for this patient’s pathology to be attributable to cocaine use or something such as tricyclic antidepressant toxicity without notable findings on her ECG (such as T-wave and ST-segment changes in the case of the former, and PR-segment prolongation or QRS-complex widening in the latter). The lack of other supporting evidence or history for a toxin ingestion or exposure makes early cyanide toxicity and carbon monoxide poisoning unlikely as well.

The patient’s vital signs did improve after receiving a broad-spectrum antibiotic and IV fluids, which could support a diagnosis of sepsis related to an unidentified infection. Infectious possibilities include a urinary tract infection or other etiologies that would cause chest pain, such as mediastinitis, septic arthritis, endocarditis, herpes zoster with or without bacterial cellulitis, and pneumonia with or without empyema. Left-sided chest wall pain is not likely to involve a bony joint, and with a clear CXR and no other symptoms or physical findings to support these diagnoses, the potentially positive urinalysis is the only indication of infection. A urinary tract infection is not much of a diagnostic dilemma, and one would expect a decreased diastolic pressure in sepsis from vasodilation rather than isolated systolic hypotension. Infectious etiologies are, therefore, removed from the differential diagnosis.

Gastrointestinal (GI) pathologies such as esophageal rupture, peptic ulcer disease with hemorrhage, and pancreatitis can cause some of the symptomatology, including chest pain, with which the patient presented, although again, there are no findings on her radiographs to indicate any of these. Her lipase is not elevated, and she has no history of chronic pancreatitis or alcohol use that might indicate a burnt-out pancreas incapable of causing an elevated lipase despite the presence of acute pancreatitis or pancreatic trauma. Although her smoking history does put her at higher risk of *Helicobacter pylori* infection, she is not on any medications that would predispose her to ulcer formation, and overall, a primary GI etiology to her presentation is unlikely.

What is left then are the primarily cardiovascular and traumatic diagnoses that could lead to her presentation. Even within these categories, the differential diagnosis for chest pain is vast and not significantly narrowed by the additional complaint of dyspnea. Incorporating the findings of hypotension and clear lungs, however, allows refinement of the differential to a more manageable list that permits directed intervention and stabilization.

As previously noted, a relatively bland ECG and no mention of palpitations or other arrhythmias will strike acute coronary syndrome and unstable arrhythmias from the differential diagnosis. Although not 100% sensitive, a clear CXR without shift in the cardiac silhouette or sign of effusion makes a hemodynamically significant hemothorax or pneumothorax unlikely; so these will come off the differential diagnosis as well. Lower rib fractures could cause liver or splenic lacerations, but to cause hypotension these entities should cause a more pronounced anemia, and they do not really account for the pronounced cardiac lab abnormalities and normal ALT. What remains on my differential diagnosis then are cardiomyopathy, PE, cardiac tamponade, and aortic injury. Despite the elevated cardiac markers, isolated cardiomyopathy without an inciting factor is not enough of a diagnosis. There are no signs indicative of a sudden decompensated heart failure due to chronic illness, such as pulmonary edema for left heart failure, or hypoxia due to severe pulmonary hypertension leading to right heart failure with lower extremity edema or hepatic congestion.

Aortic injury can be associated with secondary coronary ischemia or even tamponade, but her traumatic event is not consistent with the high shear stress that causes aortic trauma, which typically occurs with high-speed motor vehicle collisions or falls from great height. Both PE and cardiac tamponade can be associated with an acute-onset obstructive shock, with an elevated D-dimer, troponin, and natriuretic peptide, with hepatic congestion and elevated transaminases, with chest pain and dyspnea, and with clear lungs. Looking specifically at the transaminases, the elevation of AST without ALT leans more toward injury of a non-hepatic tissue, such as the cardiac muscle, rather than simple hepatic congestion. Returning to the provided ECG with these two diagnoses in mind, electrical alternans is noted to be present. Thus, considering all the above factors and in the setting of an admittedly unusual but definite blunt trauma, the most likely diagnosis is cardiac tamponade, with a point-of-care ultrasound being the diagnostic test of choice.

## CASE OUTCOME (DR. WYNNE)

The patient was sent for a computed tomography, which showed moderate hemopericardium with no PE or aortic dissection. She then had a formal echocardiogram in the ED, which showed a moderate to large pericardial effusion with impending cardiac tamponade. The patient was ultimately transferred to a trauma center and taken immediately to the operating room on arrival. Sternotomy and pericardial window were performed at which time 300 milliliters of blood was evacuated. The surgical team discovered that the apex of the heart had ruptured, and this was repaired intraoperatively.

**Image 3.**
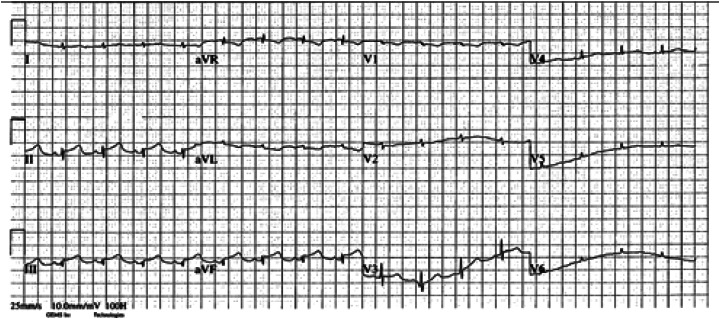
Postoperative electrocardiogram of a 54-year-old woman with chest pain.

The following day, the patient had an ECG (Image 3) that showed an inferior ST-segment elevation myocardial infarction. Repeat transthoracic echocardiogram showed apical hypokinesis. Coronary angiography done the same day showed a stumped left anterior descending artery, likely secondary to the recent surgical repair. Her postoperative course was complicated by multiple pneumothoraces, but she was ultimately discharged from the hospital several weeks later.

## RESIDENT DISCUSSION

Cardiac tamponade remains a relatively rare but striking presentation of obstructive circulatory shock caused by an accumulating pericardial effusion. The pericardial effusion can be a result of multiple etiologies including blunt trauma (as in our patient), penetrating trauma, malignancy, infection (such as tuberculosis), autoimmune conditions, uremia, and others.^2^^,^^3^

Physiologically, cardiac tamponade is more appropriately considered as a spectrum of disease rather than simply as being present or absent.^4^ Initially, the patient will have a pericardial effusion without physiologic signs. With accumulation of this effusion, there will be an increase in the pressure exerted by the pericardium on the cardiac chambers. Patients are often asymptomatic at this stage but are considered at risk for tamponade.^5^ The rate of accumulation of the fluid determines the volume threshold at which symptomatic cardiac tamponade occurs.^3^^,^^5^ Quickly accumulating effusions, such as those seen in blunt or penetrating cardiac injuries, will lead to tamponade physiology developing at lower effusion volumes. This is due to the inability of the fibrous pericardial sac to stretch rapidly.^5^ In the case of subacute or chronic effusions, the pericardial sac can compensate over time, leading to effusion volumes as high as one liter prior to the development of cardiac tamponade.^5^

Once the pericardial pressure exceeds the right atrial pressure, venous inflow from the vena cava is impaired due to diastolic collapse of the right atrium. This can initially be overcome with increased central venous pressure through fluid resuscitation.^3^ Eventually, increasing pericardial pressure leads to right atrial collapse in systole (sensitive for cardiac tamponade) and ventricular collapse in diastole (specific for cardiac tamponade).^6^^,^^7^ Next, the pericardial effusion begins to overcome diastolic filling pressures of all four chambers. This leads to increased interventricular dependence, the state in which all four chambers must share a fixed intrapericardial volume.^8^ During inspiration, decreased intrathoracic pressure and subsequent decreased pulmonary vascular resistance leads to increased filling of right-sided chambers with consequent decreased filling of left chambers in this fixed volume. The opposite occurs during expiration in which left-sided chambers are filled more so than the right-sided chambers due to increased resistance encountered by the right-sided chambers. This phenomenon leads to respiratory variation in cardiac output, known as pulsus paradoxus (defined by a decline by 10 mm Hg or greater in systolic blood pressure during inspiration).^8^^,^^9^

Cardiac output is initially maintained by increases in heart rate as well as increased systemic vascular resistance by endogenous catecholamines. These patients are often hypertensive early in their course;^5^ however, decreased stroke volumes from decreased chamber filling eventually leads to hypotension. Narrow pulse pressure remains a physiologic hallmark of cardiac tamponade due to worsening cardiac output. Eventually, pressures of the four cardiac chambers equalize and pericardial pressures begin to increase exponentially. A critical volume is reached after which the significantly decreased cardiac output is unable to perfuse the coronary arteries sufficiently, known as “last drop phenomenon.” Patients often then have a vagal response and develop cardiac arrest due to coronary arterial hypoperfusion.^5^

Typical symptoms of cardiac tamponade include dyspnea, chest discomfort or pain, and tachypnea.^5^ Patients with late-stage cardiac tamponade may show clinical signs of circulatory shock such as altered mental status and cool extremities. Patients will often have clear lung sounds if no other pathologic process is present. Subacute forms of cardiac tamponade will show signs of right heart failure, such as lower extremity edema and jugular venous distension.^3^^,^^5^ Beck triad (jugular venous distension, muffled heart sounds, and hypotension) has often been taught in medical schools to clinically diagnose cardiac tamponade,^10^ but recent research suggests that these findings, separate or in combination, may not sufficiently exclude or rule in cardiac tamponade.^11^^,^^12^ Electrocardiogram findings such as low QRS-complex voltage and electrical alternans can suggest cardiac tamponade but are neither sensitive nor specific to the condition.^13^

As clinical diagnosis can be inaccurate, point-of-care or formal echocardiogram should be obtained in patients with undifferentiated dyspnea with concern for possible cardiac tamponade.^10^^,^^14^ The presence of a pericardial effusion should warrant further investigation. The size of the effusion alone will not determine where a patient falls on the spectrum of illness of cardiac tamponade. Right atrial collapse during systole is sensitive for cardiac tamponade while right ventricular diastolic collapse and left atrial systolic collapse are specific.^5^^–^^7^ A plethoric inferior vena cava without respiratory variation, although nonspecific, further supports a diagnosis of cardiac tamponade, while its absence makes it unlikely. Other sonographic findings include mitral inflow velocity respiratory variation (also known as sonographic pulsus paradoxus), and hepatic flow reversal (measured by pulsed wave Doppler).^6^^,^^7^^,^^15^

Management of the obstructive circulatory shock caused by cardiac tamponade focuses on removal of the pericardial effusion.^10^^,^^14^ Prior to removal of fluid, patients can be initially stabilized with fluid resuscitation if they appear hypovolemic.^3^ Often, these patients have maximum intrinsic catecholamine stimulation, and further inotropic support does not lead to improved hemodynamics.^5^ Positive pressure ventilation should be used cautiously and only if absolutely necessary due to the risk of further decreases in venous return from increased intrathoracic pressure.^3^ Removal of the pericardial effusion can be accomplished in several ways. Bedside pericardiocentesis can be performed in the setting of impending or ongoing cardiac arrest or persistent hypotension despite fluid resuscitation and vasopressor use.^5^^,^^10^^,^^14^ Patients with purulent effusions or effusions in the setting of trauma can have pericardial drain placement done in the operating room, angiography suite or by interventional radiology. In patients with traumatic arrests, thoracotomy with pericardiotomy can be done to rapidly decompress the pericardium and assess for other traumatic injuries of the thorax.^16^

## FINAL DIAGNOSIS

Cardiac tamponade from ventricular apex rupture in the setting of blunt cardiac trauma

## KEY POINTS

1. Patients who present early in the course of cardiac tamponade will often be tachycardic and hypertensive with narrow pulse pressures. Hypotension and signs of end-organ failure are late findings.
2. The pressure, not the volume, of the pericardial effusion will determine when a patient develops clinical evidence of cardiac tamponade. A slower accumulation rate of pericardial fluid will allow greater effusion volumes prior to signs and symptoms appearing.
3. Consider early point-of-care echocardiography in patients with undifferentiated dyspnea who have no obvious pulmonary etiology.
